# Efficacy of soluble lansoprazole-impregnated beta-tricalcium phosphate for bone regeneration

**DOI:** 10.1038/s41598-022-25184-4

**Published:** 2022-11-29

**Authors:** Kenichi Mishima, Yuka Tsukagoshi Okabe, Masaaki Mizuno, Kinji Ohno, Hiroshi Kitoh, Shiro Imagama

**Affiliations:** 1 Department of Orthopaedic Surgery, Nagoya University Graduate School of Medicine, 65 Tsurumai-Cho, Showa-Ku, Nagoya, Aichi 466-8550 Japan; 2 Center for Advanced Medicine and Clinical Research, Nagoya University Hospital, 65 Tsurumai-Cho, Showa-Ku, Nagoya, Aichi 466-8550 Japan; 3 Department of Orthopaedic Surgery, Aichi Children’s Health and Medical Center, 7-426 Morioka-cho, Obu, Aichi 474-8710 Japan; 4 Division of Neurogenetics, Center for Neurological Diseases and Cancer, Nagoya University Graduate School of Medicine, 65 Tsurumai-cho, Showa-ku, Nagoya, Aichi 466-8550 Japan

**Keywords:** Drug screening, Translational research, Biomaterials

## Abstract

The proton pump inhibitor lansoprazole has been previously identified to upregulate the expression and transcriptional activity of runt-related transcription factor 2 (Runx2) that promotes lineage commitment and differentiation of osteoprogenitor cells. We could not elicit the expected efficacy of insoluble lansoprazole in enhancing osteogenesis when combined with beta-tricalcium phosphate (β-TCP) bone substitutes. This study aimed to evaluate the effects of soluble lansoprazole on in vitro osteoblastogenesis and new bone formation in vivo. Commercially available human mesenchymal stem cells or patient-derived bone marrow-derived stromal cells were treated with 20 µM of soluble lansoprazole at the beginning of osteogenic induction. Soluble lansoprazole-impregnated β-TCP materials were embedded in the cortical bone defect model of rabbits. Rabbits were sacrificed four weeks postoperatively and undecalcified bone specimens were prepared for evaluation of intra-material new bone formation. Only a 1-day treatment with soluble lansoprazole facilitated osteoblastic differentiation and matrix calcium deposition when added to undifferentiated human mesenchymal stromal cells at the beginning of the osteogenic differentiation. Soluble lansoprazole dose-dependently accelerated intra-material new bone formation when being impregnated with porous β-TCP artificial bones. Local use of soluble lansoprazole can be applicable for fracture and bone defect repair when combined with porous β-TCP scaffolds.

## Introduction

Using drug repositioning approaches, lansoprazole, a proton pump inhibitor commonly prescribed for the treatment of acid-related diseases, has been previously identified to upregulate the expression and transcriptional activity of runt-related transcription factor 2 (Runx2)^1^, a master transcriptional modulator of osteoblastic differentiation that plays a fundamental role in cell fate commitment and differentiation of mesenchymal stem cells to osteoblast-lineage cells^2,3^. Lansoprazole facilitated osteoblastic differentiation in undifferentiated human mesenchymal stromal cells and accelerated the physiological processes of fracture healing in rodents^1^. In-depth exploration of the mechanism of action by using *in cellulo* and in vitro protein ubiquitination assay and Western blotting analysis of the phosphorylation levels of the bone morphogenetic protein (BMP) signaling-associated molecules revealed that lansoprazole induced lysine 63-linked autopolyubiquitination of tumor necrosis factor receptor-associated factor 6 (TRAF6), an effector molecule downstream of BMP receptors, and activated noncanonical BMP-transforming growth factor-β-activated kinase 1 (TAK1)-p38 mitogen‑activated protein kinase (MAPK) signaling. Based on the optimal range of lansoprazole concentrations (20–40 µM), previously determined through a set of our in vitro studies^1^, a dosage amount of lansoprazole necessary to elicit the effects of osteogenesis promotion in vivo was estimated to be tenfold to 20-fold higher than a regular adult dose, which has been regarded as a crucial bottleneck for its systemic administration to humans. Hence, we have undertaken developmental research on lansoprazole-containing artificial bones to evaluate the applicability of its local administration. As lansoprazole in bulk powder is a water-insoluble compound^4^, it needs to be dissolved in dimethyl sulfoxide (DMSO) before loading it into calcium phosphate-based materials. In an attempt to realize the clinical application of lansoprazole for bone regeneration, whether lansoprazole could promote local bone formation when combined with beta-tricalcium phosphate (β-TCP) bone substitutes was sought using a rabbit cortical bone defect model. However, the expected efficacy in enhancing bone regeneration within the materials was not proved because lansoprazole-impregnated artificial bones, which had been prepared by immersion of β-TCP materials in a 250 mM or a 500 mM of lansoprazole solution in DMSO, were surrounded with dense fibrogranulomatous tissues after being embedded in bones. This phenomenon was speculated to occur probably because the sustained release of insoluble lansoprazole that was synchronized with the resorption of β-TCP caused the retention of extremely higher concentrations of lansoprazole in the interface between the host bones and the materials, leading to extensive local tissue toxicity. Meanwhile, neither therapeutic efficacy on intra-material bone regeneration nor material encapsulation with fibrogranulomatous tissues was observed when using lower concentrations of lansoprazole solution.

A water-soluble form of lansoprazole has already been developed and used in clinical settings. Thus, it was supposed that one could untangle issues of this solubility-derived toxicity and a relatively narrow therapeutic window by using that. However, uncertainty existed as to whether short-term treatment with soluble lansoprazole could facilitate osteoblastic differentiation of undifferentiated mesenchymal stromal cells and the formation of calcified tissues. DMSO has been widely used for cryopreservation of cells and as an organic solvent for water-insoluble compounds in various biomedical fields^5^. Despite having been classified by the FDA as the safest category of solvent with low toxic potential^6^, DMSO has been shown to affect various cellular functions and processes including growth, differentiation, and apoptosis^7–10^. A recent omics study analyzing the DNA methylation profiles, transcriptome, and proteome of several DMSO-exposed tissues revealed that even lower concentrations of DMSO caused considerable changes in microRNAs and alterations in the epigenetic landscape, concluding that use of DMSO should be avoided where possible^11^. This is one reason why we decided to use soluble lansoprazole for the development of lansoprazole-containing bone substitutes.

The purpose of the present study is to assess the efficacy and safety of soluble lansoprazole-impregnated artificial bones by using a rabbit cortical bone defect model.

## Methods

### Culture medium and reagent

The growth medium consisted of Dulbecco’s modified Eagle’s medium (Sigma-Aldrich, St. Louis, MO) supplemented with 100 unit/mL penicillin, 100 µg/mL streptomycin, 0.75 μg/mL fungizone (Gibco, Grand Island, NY), and 10% heat-inactivated fetal calf serum (Thermo Fisher Scientific, Waltham, MA). The osteogenic differentiation medium consisted of the growth medium supplemented with 10^−7^ M Dexamethasone (Sigma-Aldrich), 10 mM Glycerol-2-phosphate disodium salt hydrate (Sigma-Aldrich), 200 μM Ascorbic acid (FUJIFILM Wako Pure Chemical Corp., Ltd., Osaka, Japan). A water-soluble form of lansoprazole was purchased from Takeda Pharmaceutical Company Limited (Osaka, Japan). Since we previously identified that 20 µM of lansoprazole exerted the maximum effect of upregulating Runx2 and terminal osteoblastic differentiation without apparent cytotoxicity in vitro, we adopted a final concentration of 20 µM as a default one of soluble lansoprazole in this study.

### Cell culture of commercially available human mesenchymal stem cells

Human bone marrow-derived mesenchymal stem cells (MSCs) were purchased from Lonza (Basel, Switzerland) and subcultured in the growth medium up to passage 5. The medium was changed every three days. To induce in vitro osteogenic differentiation, passaged cells were seeded into each well of the assay plate at a cell density of 3 × 10^5^ cells/cm^2^ and cultured in the osteogenic medium in which soluble lansoprazole was added to yield a final concentration of 20 μM (day 0). Cells were treated with soluble lansoprazole for one, three, or five days, followed by culture in the osteogenic medium without lansoprazole until day 14. The activity of alkaline phosphatase (ALP) in cell lysates was measured on day 5. Expression levels of *Runx2* mRNA and osteocalcin concentrations in cell lysates were measured on day 14.

### Cell culture of patient-derived bone marrow aspirates

After institutional review board approval and acquisition of written informed consent from an otherwise healthy child with acetabular dysplasia aged 5 years and her caregivers, a 20-mL of bone marrow aspirate was obtained from the patient at the time of a reconstructive pelvic osteotomy. All methods were carried out in accordance with relevant guidelines and regulations. A mononuclear cells-enriched fraction was extracted from the aspirate by density gradient centrifugation with the use of Ficoll-Paque (GE Healthcare Life Sciences, Pittsburgh, PA) and cultured in the growth medium (day 0). Nonadherent hematopoietic cells were carefully removed by extensive washing on days 3 and 6, and resultant plastic-adherent cells were detached and reseeded into each well of the assay plate at a cell density of 1 × 10^4^ cells/cm^2^ on day 7. Part of the detached cells was subjected to flow cytometric analysis on the same day, and the rest was osteogenically induced with the osteogenic medium containing 20 µM of soluble lansoprazole for 24 h, followed by culture in the lansoprazole-free osteogenic medium until day 28. Osteocalcin concentrations in cell lysates were measured on day 21. For the mineralized nodule assay, alizarin red staining was performed on day 28.

### ALP activity and osteocalcin enzyme-linked immunosorbent assay

Cells were washed with phosphate-buffered saline (PBS) solution twice and lysed in 1% Triton X-100 (Sigma-Aldrich). ALP activities in cell lysates were determined by colorimetric analysis using ρ-nitrophenyl phosphate (PNPP) as substrates. Total protein contents in cell lysates were determined by the Pierce 660-nm Protein Assay Kit (Thermo Fisher Scientific) using bovine serum albumin as the standard. Osteocalcin concentrations in cell lysates were measured using human intact osteocalcin EIA kit (Biomedical Technologies Inc., Stoughton, MA), according to the manufacturer’s instructions. The ALP activity and osteocalcin concentration were normalized to the total protein content of each lysate.

### Alizarin red S staining

Cells were washed with PBS solution twice, fixed with 4% paraformaldehyde, and stained with 0.1% alizarin red S solution (pH 6.3) for 20 min at room temperature and rinsed with distilled water three times. Microscopy images of the stained cells were acquired using BZ-9000 (Keyence Corp., Osaka, Japan).

### Flow cytometric analysis

After washing, cells were stained with various combinations of monoclonal antibodies including Pacific Blue-conjugated anti-CD45, fluorescein isothiocyanate (FITC)-conjugated anti-CD90, allophycocyanin (APC)-conjugated anti-CD73, Phycoerythrin (PE)-conjugated anti-CD105 from Beckman Coulter (Fullerton, CA), and isotype control from BioLegend (SanDiego, CA). The stained cells were analyzed with MACSQuant analyzer (Miltenyi Biotec, Bergisch Gladbach, Germany), and the acquired data were analyzed with FlowJo software (Tree Star, Inc., Ashland, OR).

### Preparation of soluble lansoprazole-impregnated artificial bones

We used a high-porosity unidirectional porous β-TCP scaffold for grafting (Courtesy from Kuraray Co Ltd., Japan). Its pore size is 35–250 µm and its porosity is 57 ± 5%. Trapezoidal-shaped ones were immersed in each of 20 µM, 200 µM, or 2 mM of lansoprazole solution in water for three minutes, followed by a rinse in PBS solution for one minute.

### Rabbit cortical bone defect model

All animal care and handling were approved by the institutional animal care and use committee and performed in accordance with the ARRIVE guidelines. All surgical procedures were conducted under usual sterile conditions. Thirteen-week-old male Japanese white rabbits (approximately 2.5 kg of body weight) were anesthetized by intramuscular injection of ketamine and xylazine for rapid induction and inhaled isoflurane for maintenance of anesthesia. The skin, fascia, and periosteum over the proximal tibia of both hindlimbs were incised and retracted using a retractor. A rectangular bone defect accommodating a 5 mm-wide, 7 mm-long, and 5 mm-depth bone substitute was created in the anterolateral cortex of the proximal tibia using a dedicated template and a micro-burr. The defect was flushed with sterile saline to remove residual cortical bone debris. Each of the experimental and control scaffolds was closely fitted into the defect in the left and right hindlimbs, respectively. The defect was covered with the muscle and the skin was closed in layers. The animals were free to mobilize and weight-bear immediately after the procedure as tolerated. All rabbits were sacrificed, and the proximal tibias of both hindlimbs were harvested at four weeks postoperatively.

### Bone histological analysis

Bone specimens were sequentially dehydrated with 70% ethanol, 90% ethanol, and acetone. The specimens were then embedded without decalcification in methyl methacrylate (MMA). Six-µm mid-sagittal sections of the proximal tibias were prepared with a microtome and stained them with Villanueva Goldner staining. For analysis of bone regeneration within the porous materials, the surface area of newly formed bones that were stained green was automatically measured using a computer-associated image analyzer software WinROOF (Mitani Co Ltd, Japan), and then two area ratios were defined, one was the area of newly formed bones within the entire material (total bone area, TBA) per that of the entire material (total material area, TMA), and another was the area of newly formed bones within the specific area of the material embedded in the cortical bone area (partial bone area, PBA) per that of the specific area of the material embedded in the cortical bone area (partial material area, PMA). The latter ratio PBA/PMA was used for quantitative assessment of the cortical healing that could partly reflect the osteoconductive ability of the materials.

### Radiographic evaluation

Anteroposterior and lateral radiographs of all harvested tibias were taken using a soft X-ray film shooting apparatus (Softex Co Ltd, Tokyo, Japan), and microcomputed tomography (µ-CT) was performed on all tibias using inspeXio SMX-90CT Plus (Shimadzu Co Ltd, Kyoto, Japan). The X-rays and µ-CT images were examined to qualitatively speculate about the fibrogranulomatous tissue formation around bone substitutes and the state of material absorption.

### Statistical analysis

All data are presented as the mean and standard deviation (SD). All statistical analyses were performed with IBM SPSS statistics 28 (SPSS Inc., Chicago, IL, USA). Statistical differences between the two groups were determined by the unpaired *t*-test. A one-way ANOVA with post-hoc Tukey’s test was applied to determine whether there were any statistically significant differences among the means of three or more independent groups. We evaluated dose responses of lansoprazole using the Jonckheere-Terpstra trend test over the indicated concentration range of soluble lansoprazole solution. The Jonckheere-Terpstra test is a nonparametric test for ordered differences that statistically determines the trend of dose–response effects. Although one-way ANOVA usually gives more stringent values than the Jonckheere-Terpstra trend test, one-way ANOVA cannot be applied to estimate dose responses. A *p*-value < 0.05 was considered statistically significant.

### Ethics approval

All animal experiments were performed in accordance with institutional guidelines and were approved by the Nagoya University Institutional Animal Care and Use Committee. The extraction of bone marrow aspirates was approved by the Nagoya University Hospital Institutional Review Board. All bone marrow aspirates were harvested from the iliac crest during Salter innominate osteotomy indicated for the treatment of developmental dysplasia of the hip.

## Results

### Insoluble lansoprazole-impregnated artificial bones did not demonstrate the facilitation of intra-material bone regeneration

**Figure 1:**
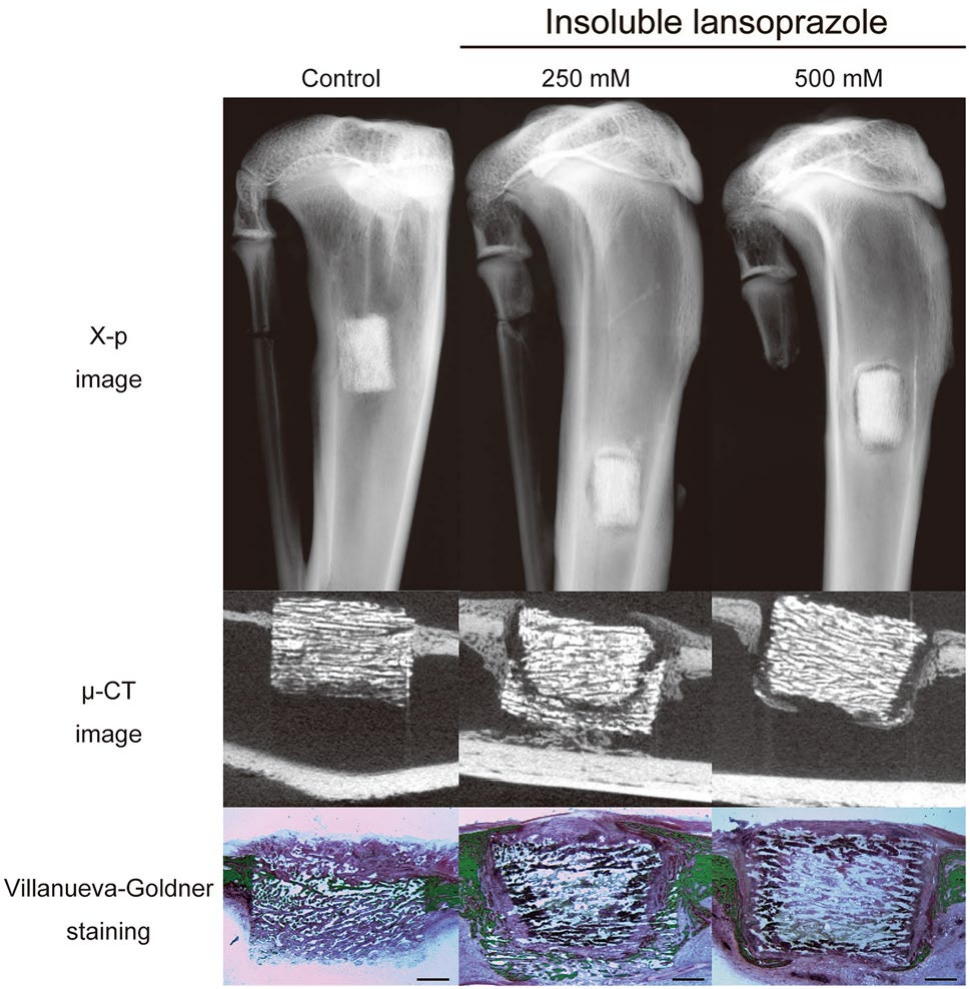
Radiographic, µ-CT, and histological images of insoluble lansoprazole-impregnated artificial bones embedded for four weeks in a rabbit cortical bone defect model. Note that radiolucent areas around lansoprazole-impregnated materials correspond to dense fibrogranulomatous tissues on histology (Villanueva-Goldner staining). In contrast to the control one, the insides of the experimental materials surrounded with the fibrogranulomatous tissues are devoid of newly-formed bones (green). Scale bars, 1 mm.

Insoluble lansoprazole-impregnated artificial bones, which had been prepared by immersion of β-TCP materials in a 250 mM or a 500 mM of lansoprazole solution in DMSO, were surrounded with dense fibrogranulomatous tissues after being embedded in bones, thereby hindering cell migration into them (Fig. 1). The concentrations of the lansoprazole solution were set based on a preliminary experiment to estimate the release profile under a simulated bone defect condition (Supplementary Fig. 1). Meanwhile, neither therapeutic efficacy on intra-material bone regeneration nor material encapsulation with fibrogranulomatous tissues was observed when using lower concentrations (10 mM, 25 mM, and 40 mM) of lansoprazole solution in DMSO for the preparation of the scaffolds (Supplementary Figs. 2 and 3).

### Short-term treatment with soluble lansoprazole facilitates osteoblastic differentiation of human MSCs

**Figure 2:**
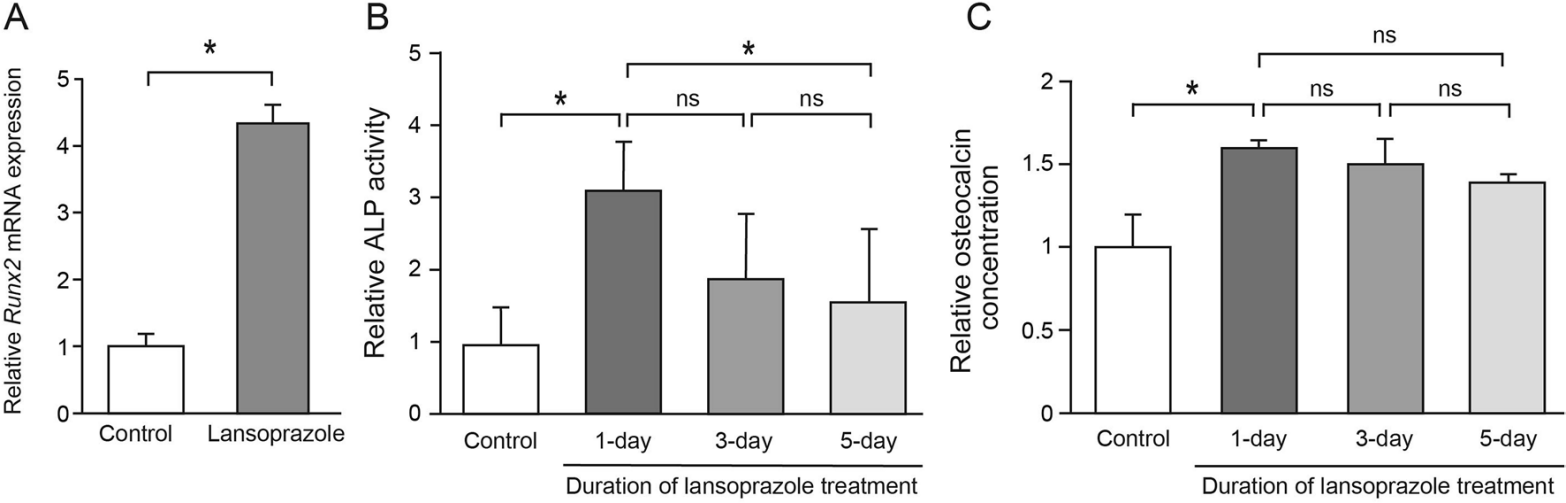
Short-term treatment with soluble lansoprazole facilitates osteoblastic differentiation of human mesenchymal stem cells. (**A**) Expression levels of endogenous *Runx2* mRNA in soluble lansoprazole-treated commercially available human MSCs. Cells were treated with soluble lansoprazole for 1 day at the beginning of osteogenic induction. The relative expression levels were normalized to the mean of the control. (**B**) ALP activity of cell lysates of soluble lansoprazole-treated commercially available human MSCs. Cells were cultured in the osteogenic medium for 14 days and treated with soluble lansoprazole for one, three, or five days at the beginning of osteogenic induction (day 0). The ALP activities were determined on day 5. The relative activity was normalized to the mean of the control. (**C**) Osteocalcin concentration of cell lysates of soluble lansoprazole-treated commercially available human MSCs. Cells were cultured in the osteogenic medium for 14 days and treated with soluble lansoprazole for one, three, or five days at the beginning of osteogenic induction (day 0). The osteocalcin concentrations were determined on day 14. The relative concentration was normalized to the mean of the control. In (**A**–**C**), the mean and SD (**A**, *n* = 3; **B** and **C**, *n* = 6) are indicated. (**A**) *p < 0.05 by unpaired *t*-test. (**B**, **C**) *p < 0.05 by one-way ANOVA with post hoc Tukey analysis. ns, not significant.

We undertook to examine the short-term effects of soluble lansoprazole on the promotion of osteogenesis in vitro. Since we previously identified that 20 µM of lansoprazole exerted the maximum effect of upregulating Runx2 and terminal osteoblastic differentiation without apparent cytotoxicity in vitro^1^, we adopted a final concentration of 20 µM as a default one of soluble lansoprazole in this study. To examine the effects of short-term treatment with soluble lansoprazole on upregulation of Runx2 and osteoblastic differentiation, we cultured commercially available human MSCs for one day in the osteogenic medium containing 20 µM of soluble lansoprazole, and found that soluble lansoprazole significantly increased expressions of *Runx2* mRNA when added at the beginning of osteogenic induction (Fig. 2A). We also confirmed that ALP activity and osteocalcin concentration, Runx2-dependent early and late markers of osteoblastic differentiation, respectively, were also significantly elevated with the addition of soluble lansoprazole (Fig. 2B, C). Notably, as the effect of a 1-day treatment with soluble lansoprazole was more prominent than that of 3-day or 5-day treatment, we adopted the 1-day treatment as a default condition in this study.

### Short-term treatment with soluble lansoprazole enhances matrix calcium deposition of patient-derived BMSCs

**Figure 3:**
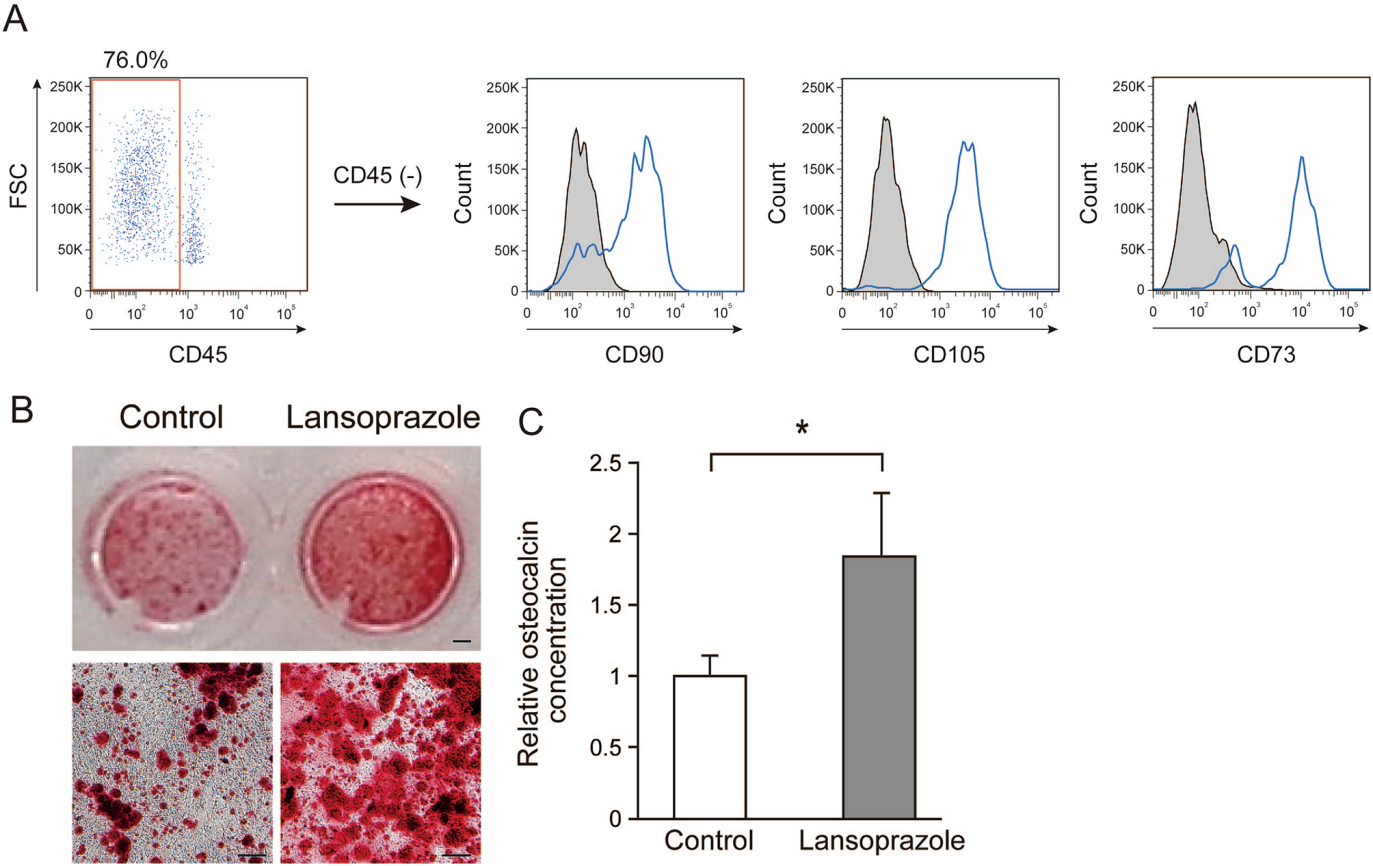
Short-term treatment with soluble lansoprazole enhances matrix calcium deposition of patient-derived bone marrow stromal cells (BMSCs). (**A**) Verification of stemness characteristics of plastic-adherent cells obtained from primary culture of patient-derived bone marrow aspirates in the growth medium. A pan-leukocyte marker CD45-negative adherent cells were evaluated for expression levels of typical MSC markers CD90, CD105, and CD73 by FACS analysis. Blue histograms show the fluorescent intensity for the indicated cell surface markers. Shadow histograms represent the fluorescent intensity obtained with the control antibodies. X-axis and Y-axis indicate fluorescent intensity and cell count, respectively. FSC, forward scatter. (**B**) Matrix calcium deposition detected by alizarin red staining in patient-derived BMSCs. Cells were treated with 20 µM of soluble lansoprazole for 1 day at the beginning of osteogenic induction and cultured for 21 days in the osteogenic medium without lansoprazole. Scale bar, 1 mm (upper panel) and 200 µm (lower panel). (**C**) Osteocalcin concentration of cell lysates of patient-derived BMSCs. Cells were treated with soluble lansoprazole for 1 day at the beginning of osteogenic induction and cultured for 14 days in the osteogenic medium without lansoprazole. The relative concentration was normalized to the mean of the control. The mean and SD (*n* = 3) are indicated. *p < 0.05 by unpaired *t*-test.

Before proceeding to embedding of soluble lansoprazole-impregnated β-TCP materials into bone defects, we assessed the effects of soluble lansoprazole in close to the transplant environment using bone marrow cells. To characterize plastic-adherent cells isolated from patient-derived bone marrow aspirates, they were subjected to flow cytometric analysis. Most adherent cells expressed CD73, CD90, and CD105 and not CD45, proving that they possess stem cell-like characteristics (Fig. 3A). We next examined the effects of soluble lansoprazole on terminal osteoblastic differentiation and mineralized matrix deposition using these patient-derived BMSCs and found that the 1-day treatment with soluble lansoprazole increased cellular expression of osteocalcin and accelerated matrix calcium deposition in osteogenically induced BMSCs (Fig. 3B, C). These results suggested that only short-term treatment with soluble lansoprazole could promote terminal osteoblastic differentiation and matrix mineralization in human primary undifferentiated BMSCs.

### Soluble lansoprazole-impregnated artificial bones demonstrate the acceleration of intra-material bone regeneration

**Figure 4:**
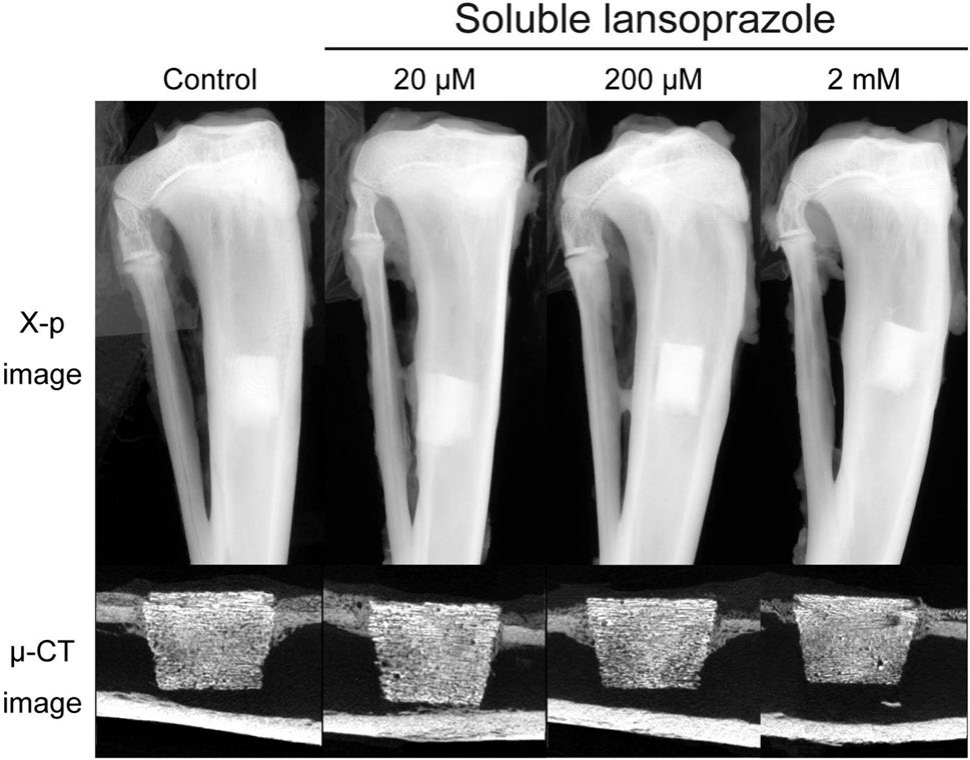
Radiographic and µ-CT images of soluble lansoprazole-impregnated artificial bones embedded for four weeks in a rabbit cortical bone defect model. In contrast to insoluble-lansoprazole ones (Fig. 1), experimental materials appeared to fuse with the host bones.

**Figure 5:**
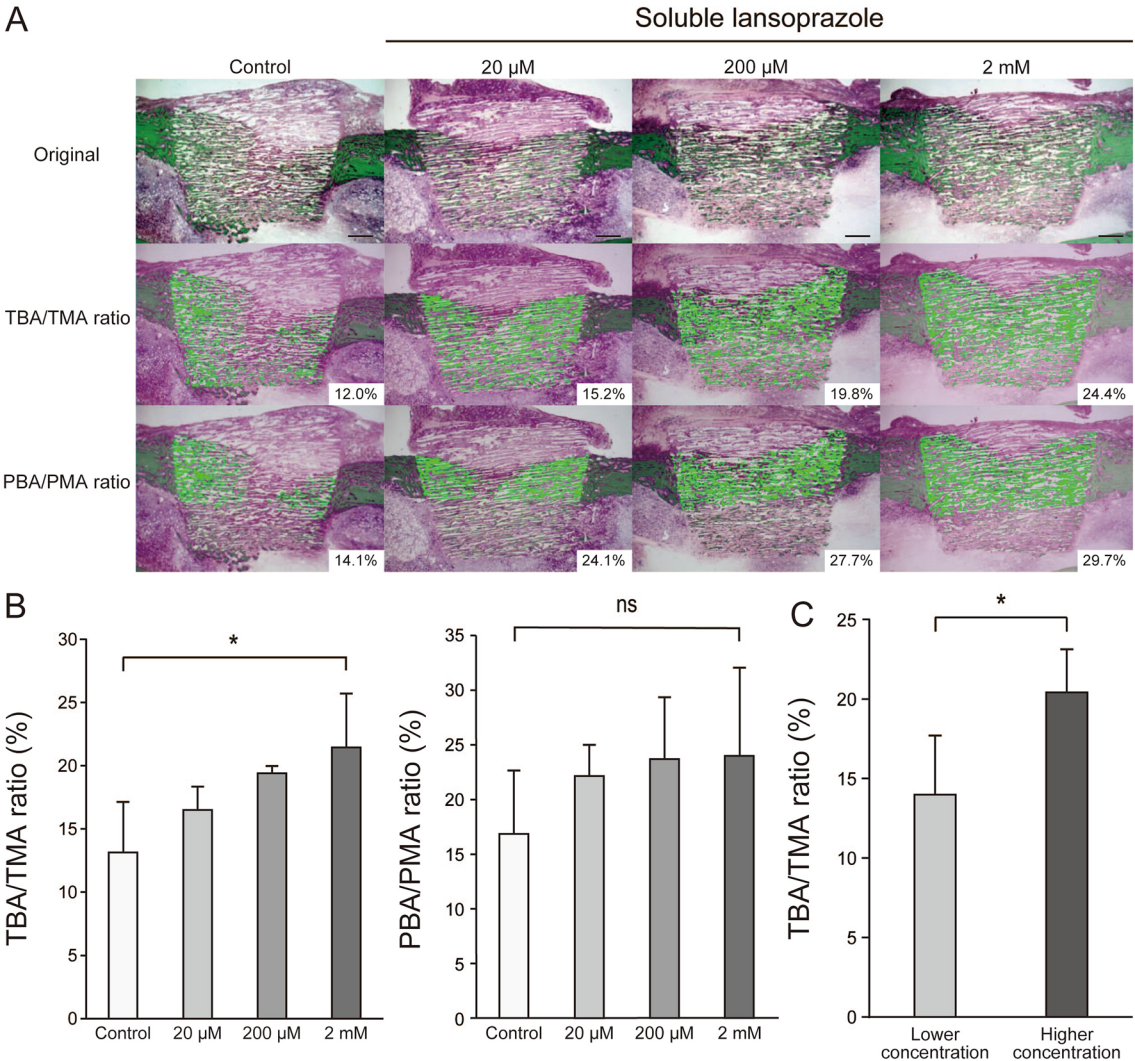
Soluble lansoprazole-impregnated artificial bones demonstrate the acceleration of intra-material bone regeneration. (**A**) Undecalcified bone histology of soluble lansoprazole-impregnated artificial bones embedded for four weeks in a rabbit cortical bone defect model (Villanueva-Goldner staining; upper panel). The surface area of newly formed bones within the materials was automatically measured using an image analyzer (WinROOF). The area of newly formed bones within the entire material (total bone area, TBA; middle panel) and that within the specific area of the material embedded in the cortical bone area (partial bone area, PBA; lower panel) are colored green. TMA, total material area; PMA, partial material area. Each of the area percentage ratios is indicated in the inset of the middle panel (TBA/TMA ratio) and the lower panel (PBA/PMA ratio). (**B**) The percentage of new bone formation within the entire material (TBA/TMA ratio, left) and the specific area of the material embedded in the cortical bone area (PBA/PMA ratio, right). (**C**) The percentage of new bone formation within the entire material (TBA/TMA ratio) of the lower concentration group (control and 20 µM) and the higher concentration group (200 µM and 2 mM). In (**B**, **C**), the mean and SD (*n* = 2 per each of three experimental subgroups and *n* = 6 for the control group) are indicated. *p < 0.05 by the Jonckheere-Terpstra tread test over the indicated concentration range of soluble lansoprazole solution (**B**) and by unpaired *t*-test (**C**). ns, not significant. Scale bars, 1 mm.

As previously noted, we could not identify the therapeutic efficacy of insoluble lansoprazole-impregnated artificial bones in a cortical bone defect model because of their encapsulation with fibrogranulomatous tissues (Fig. 1). To overcome that solubility-derived toxicity, we then impregnated porous β-TCP materials with lansoprazole solution in water and embedded them in a cortical bone defect of rabbits. In all bone specimens, no radiolucent areas around the materials or subsidence of the materials into the medullary cavity were seen on both X-rays and µ-CT images (Fig. 4). No fibrogranulomatous tissue formation was observed in the interface between the host bones and the materials on histology (Supplementary Fig. 4). Soluble lansoprazole increased two ratios of TBA/TMA and PBA/PMA in a dose-dependent manner (Fig. 5A, B). Once the specimens were grouped into two groups, a lower concentration group (control and 20 µM) and a higher concentration group (200 µM and 2 mM), the TBA/TMA ratio was significantly increased in the higher group than in the lower group (Fig. 5C).

## Discussion

The findings of the present study showed that a water-soluble form of lansoprazole, a clinically available proton pump inhibitor for intravenous infusion, upregulated Runx2 expression and enhanced matrix calcium deposition in osteogenically-induced human bone marrow-derived stromal cells. Furthermore, the osteogenic effect of a 1-day treatment with soluble lansoprazole was more prominent than that of a 3-day or a 5-day treatment. In contrast to the discouraging results of insoluble lansoprazole-containing artificial bones, soluble lansoprazole-impregnated ones demonstrated promising efficacy in bone regeneration within the materials in an animal model of the cortical bone defect. These results indicate that brief exposure of local osteoprogenitor cells to soluble lansoprazole that can be rapidly released from the materials and attenuated for a short time can be sufficient to enhance local bone regeneration without obvious cytotoxicity. The transcription factor Runx2 is essential for cell fate-decision of mesenchymal stem cells towards the osteoblast lineage, as evidenced by the phenotype of Runx2 knockout mice displaying a complete lack of bone formation and death immediately after birth due to respiratory failure^3^. Another physiological role of Runx2 is to keep osteoblast lineage cells in an immature stage. The expression of Runx2 peaks in pre-osteoblasts and immature osteoblasts and decreases in the later stage under normal physiological conditions, indicating a stimulating and an inhibitory effect of Runx2 on early osteoblastic differentiation and the transition of mature osteoblasts into osteocytes, respectively^2^. The finding that the effect of a 1-day treatment with soluble lansoprazole was more prominent than that of 3-day or 5-day treatment with respect to ALP activity (an early osteoblast marker) may reflect such a facilitating effect of lansoprazole on the early stages of osteoblastogenesis. Accordingly, it can be a realistic strategy for facilitating local bone regeneration to provide undifferentiated mesenchymal stem cells, which rapidly accumulate at the sites of bone and bone marrow injury^12^, with a short period of stimulus to upregulation of Runx2 expression and/or its transcriptional activity because once committed to the osteoblast lineage, they can be expected to differentiate mature osteoblasts and enhance calcified tissue formation under reparative conditions.

As mentioned earlier, despite a fundamental determinant of cell fate in osteoblastogenesis, sustained upregulation of Runx2 paradoxically hampers the terminal differentiation of osteoblast-lineage cells^13^. As such, prolonged exposure to lansoprazole can negatively affect bone regeneration since lansoprazole exhibits an off-label effect of Runx2 activation. Cellular ALP activity of bone marrow-derived MSCs was significantly suppressed when cultured with osteogenic medium containing lansoprazole (10–1000 µM) for a longer period (> 14 days)^14^. Thus, we decided to choose a simple method of absorption in which porous β-TCP scaffolds were only immersed in the aqueous lansoprazole solution for loading it into them. Although we are unsure of the temporal and spatial profile of lansoprazole concentration distribution within the materials after being embedded in bones, almost all amounts of lansoprazole, probably attaching to the material surface, are anticipated to release in short bursts. A previous investigation has been performed using rifampicin to compare the temporal profile of drug release from β-TCP scaffolds among three different absorption techniques, namely static, dynamic, and vacuum methods^15^. As a result, ninety percent of rifampicin was released within two days from β-TCP in which it had been loaded by the static loading method similar to ours. Based on that result, a burst release of lansoprazole was considered to occur within one or two days after the material implantation. As only 1-day treatment of soluble lansoprazole could facilitate terminal differentiation of osteoblast lineage cells under osteogenic conditions, such burst release of lansoprazole from the materials could have a beneficial effect on acceleration of bone regeneration within them.

Tricalcium phosphate-based resorbable bone substitutes have been universally used as a complement to autologous bones for the treatment of fractures and bone defects along with hydroxyapatite-based biomaterials^16^. As tricalcium phosphate ceramic exhibits superior osteoconductive ability but lacks the osteoinductive ability, it cannot be expected to provide better performance on bone regeneration compared to autologous bones. In an attempt to reinforce the osteogenic ability of artificial bones, several trials in which biomaterials are combined with various pharmacological substances such as BMPs and fibroblast growth factors (FGFs) for growth factors^17,18^, aspirin and statins for low molecular weight compounds^19,20^, and strontium for metals have been conducted^21,22^. Of them, despite having marked in vitro osteoinductive ability, combination use of BMP-2/4 or FGF-2 with biomaterials has several obstacles to commercialization including less cost-effectiveness and inevitable deactivation following sterilization^23,24^. Meanwhile, low-molecular compounds can be sterilized in advance without deactivation and their manufacturing cost is relatively inexpensive. We have thus considered simple immersion of β-TCP materials into an aqueous solution of Runx2 activator as a convenient and practical method of achieving efficacious bone regeneration because it can convert them into advanced ones with some osteoinductive ability, as exemplified here by lansoprazole. Further exploration of low-molecular compounds that can induce more Runx2 activation will be increasingly significant.

## Conclusions

Short-term treatment with soluble lansoprazole at the beginning of osteogenic induction could enhance terminal osteoblastic differentiation in undifferentiated human bone marrow-derived stromal cells. Local administration of soluble lansoprazole could be applicable in clinical settings such as fractures and bone defects when combined with β-TCP materials.

## Supplementary Information

Supplementary Information 1.

Supplementary Information 2.

Supplementary Information 3.

Supplementary Information 4.

Supplementary Information 5.

## Data Availability

The datasets used and/or analyzed during the current study are available from the corresponding author on reasonable request.

## Abbreviations

β-TCP: Beta tricalcium phosphate
MSCs: Mesenchymal stem cells
BMSCs: Bone marrow-derived stromal cells
Runx2: Runt-related transcription factor 2
BMP: Bone morphogenetic protein
TRAF6: Tumor necrosis factor receptor-associated factor 6
TAK1: Transforming growth factor-β-activated kinase 1
MAPK: Mitogen-activated protein kinase
DMSO: Dimethyl sulfoxide
ALP: Alkaline phosphatase
MMA: Methyl methacrylate
