# Supplementary material for: Efficacy of soluble lansoprazole-impregnated beta-tricalcium phosphate for bone regeneration
Source: Sci Rep. 2022 Nov 29;12:20550. doi: 10.1038/s41598-022-25184-4 (PMC9708645; doi:10.1038/s41598-022-25184-4)
Supplement: Supplementary file 1 — Supplementary Information 1. [file 41598_2022_25184_MOESM1_ESM.docx]

**Supplementary Figure legends**

**Supplementary Figure 1**

**Simulation of insoluble lansoprazole release from β-TCP scaffolds.** Cylindrical porous β-TCP test scaffolds were immersed in 3-ml of lansoprazole solution in DMSO (250 mM or 500 mM) for three minutes, followed by a rinse in 6-ml of PBS solution for one day once. The volume of the test samples was equal to that of the experimental materials. The prepared samples were immersed in 30-ml of PBS solution while stirring for one day, followed by the determination of lansoprazole concentrations in the PBS solution by ultraviolet-visible absorption spectroscopy (λmax=285.4 nm; Spectrophotometer U-3010, Hitachi High-Tech Science Corp., Tokyo, Japan) [[25](https://d.docs.live.net/17148fdbf24ba3ec/発表論文/水溶性ランソプラゾール含有人工骨/Efficacy%20of%20soluble%20lansoprazol%20for%20scientific%20reports.docx#_ENREF_25)]. We changed the PBS solution every day and measured lansoprazole concentrations in the PBS solution at the time of the replacement for five successive days (*n* = 1).

**Supplementary Figure 2**

**Radiographic, µ-CT, and histological images of lower amounts of insoluble lansoprazole-impregnated artificial bones embedded for four weeks in a rabbit cortical bone defect model.** In contrast to the results regarding higher amounts of insoluble lansoprazole-impregnated artificial bones (Figure 1), radiolucent areas and fibrogranulomatous tissues surrounding the materials were not observed. Scale bars, 1 mm.

**Supplementary Figure 3**

**Histomorphometric evaluation of lower amounts of insoluble lansoprazole-impregnated artificial bones embedded for four weeks in a rabbit cortical bone defect model.** The percentage of new bone formation within the entire material (TBA/TMA ratio, left) and the specific area of the material embedded in the cortical bone area (PBA/PMA ratio, right). The mean and SD (*n* = 3) are indicated. **p* < 0.05 by the Jonckheere-Terpstra tread test over the indicated concentration range of insoluble lansoprazole solution.

**Supplementary Figure 4**

**Magnified images of the corresponding tissue specimens shown in Figure 5.** The magnified images show the interface between the host bones and the materials. Note no fibrogranulomatous tissue formation in the interfaces.
