# Supplementary figures and images for: Efficacy of soluble lansoprazole-impregnated beta-tricalcium phosphate for bone regeneration
Source: Sci Rep. 2022 Nov 29;12:20550. doi: 10.1038/s41598-022-25184-4 (PMC9708645; doi:10.1038/s41598-022-25184-4)

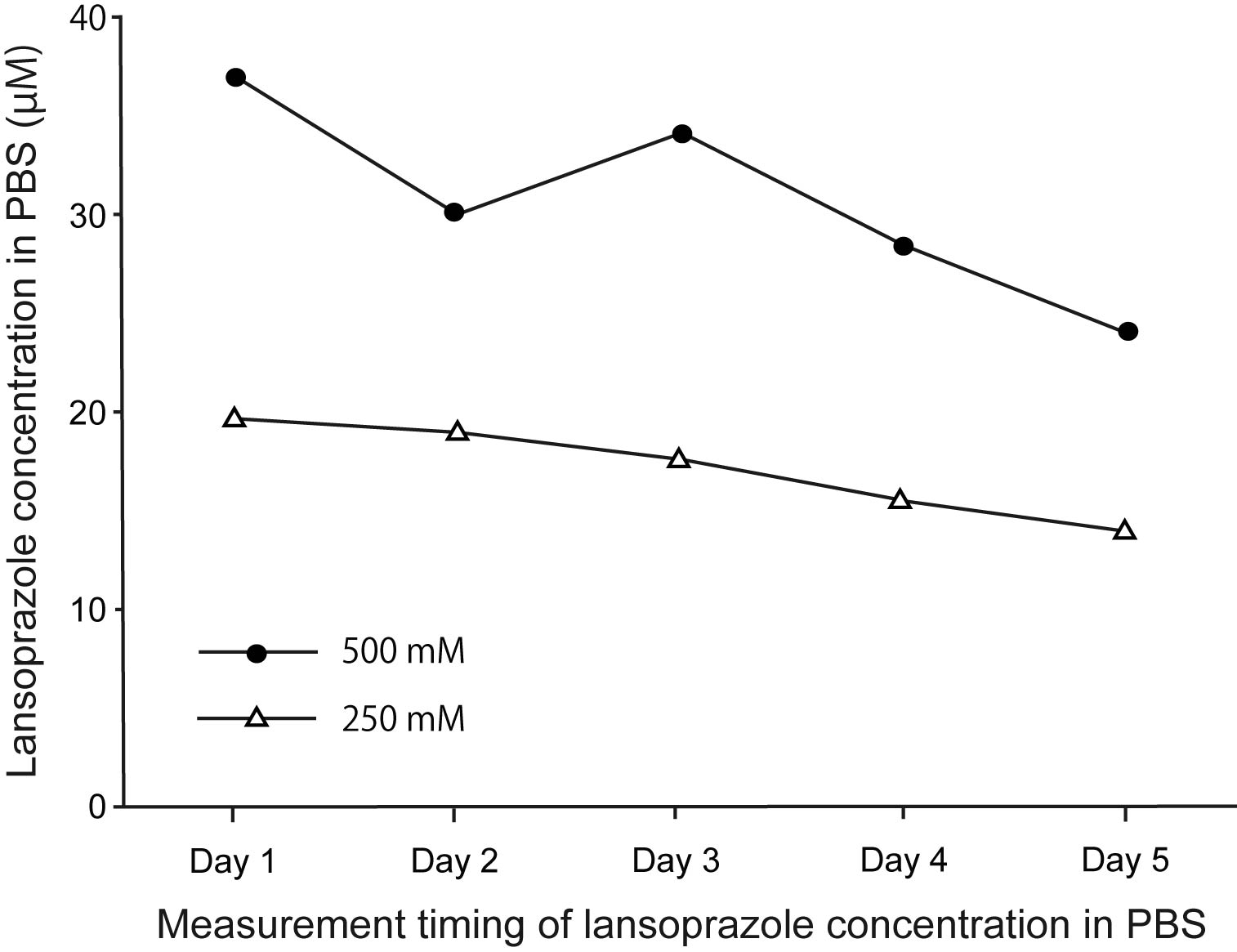

Supplement: Supplementary file 2 — Supplementary Information 2. [file 41598_2022_25184_MOESM2_ESM.jpg]

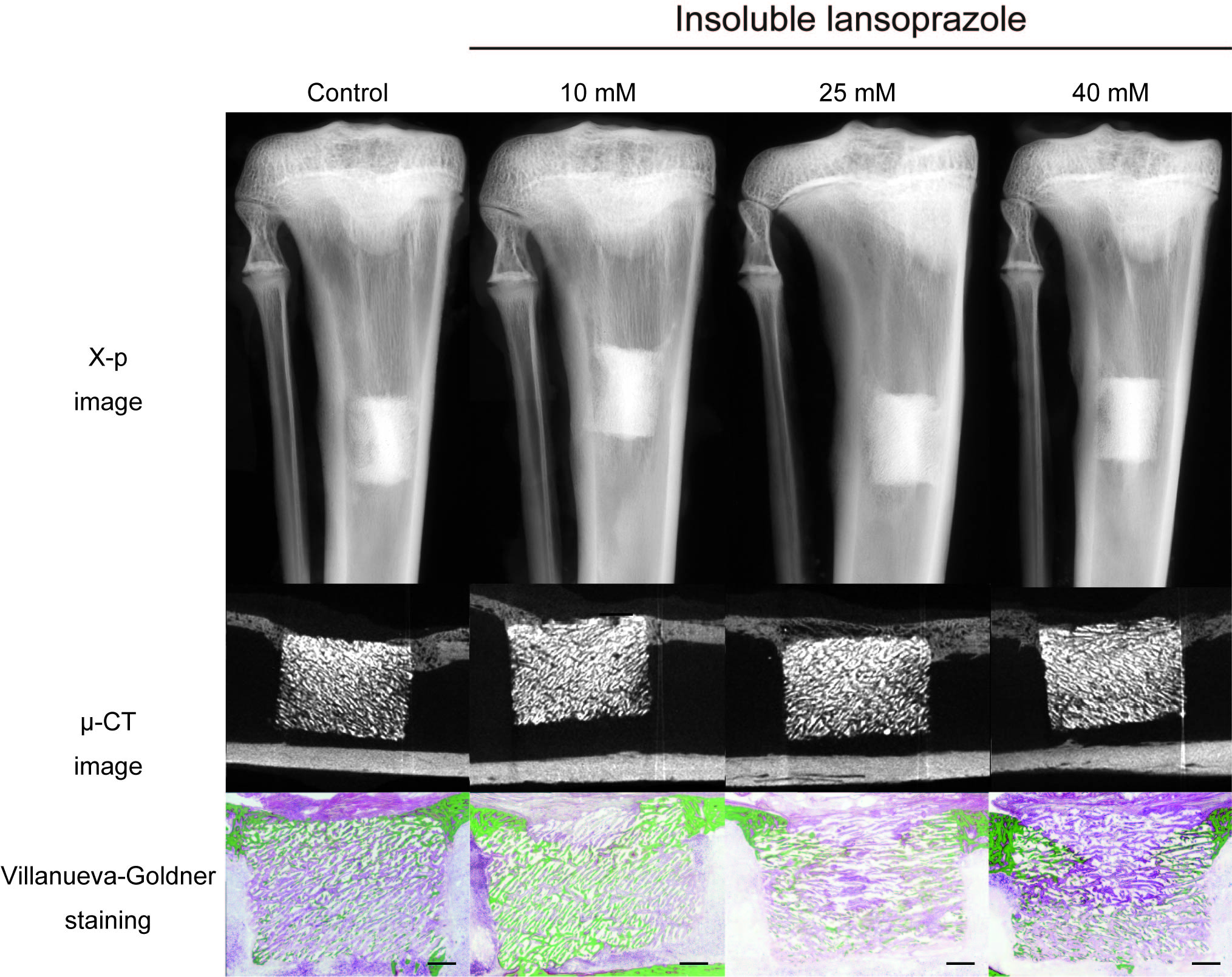

Supplement: Supplementary file 3 — Supplementary Information 3. [file 41598_2022_25184_MOESM3_ESM.jpg]

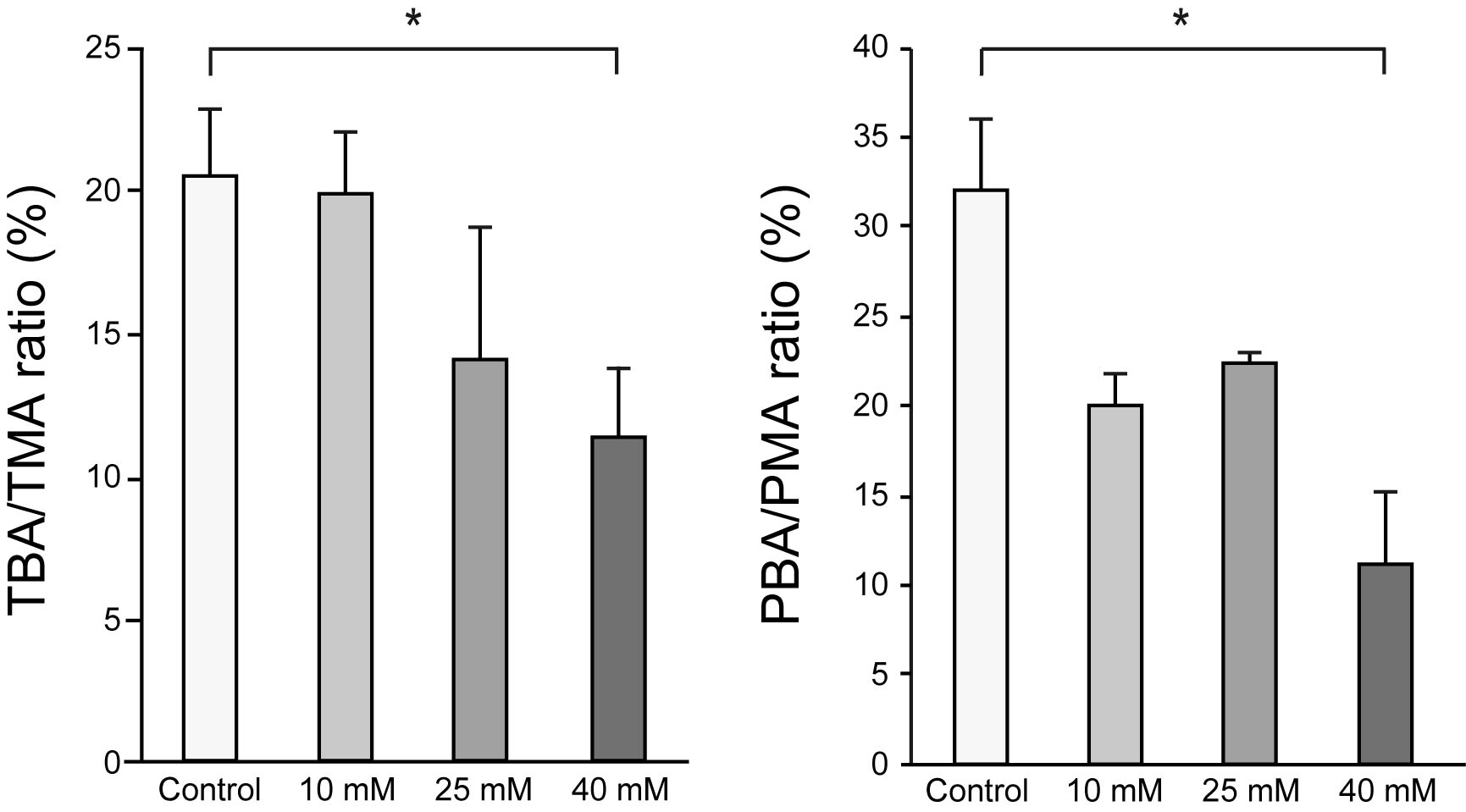

Supplement: Supplementary file 4 — Supplementary Information 4. [file 41598_2022_25184_MOESM4_ESM.jpg]

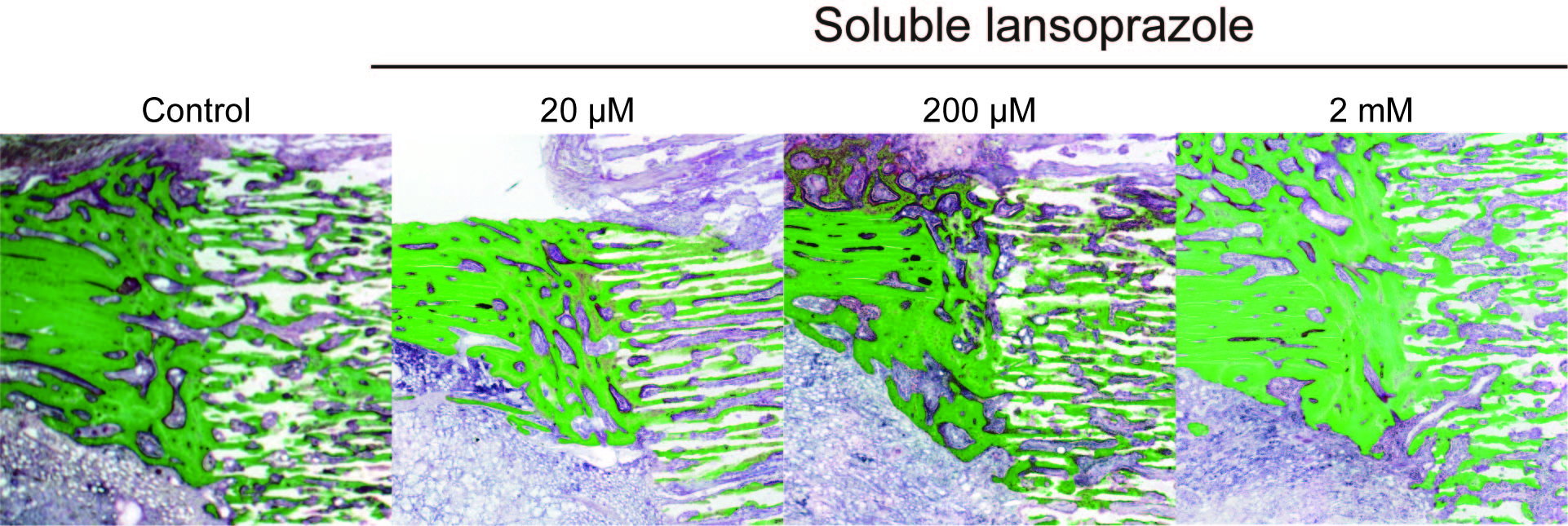

Supplement: Supplementary file 5 — Supplementary Information 5. [file 41598_2022_25184_MOESM5_ESM.jpg]
